# Tetradecanoic Acids With Anti-Virulence Properties Increase the Pathogenicity of *Pseudomonas aeruginosa* in a Murine Cutaneous Infection Model

**DOI:** 10.3389/fcimb.2020.597517

**Published:** 2021-01-27

**Authors:** Martha María Juárez-Rodríguez, Humberto Cortes-López, Rodolfo García-Contreras, Bertha González-Pedrajo, Miguel Díaz-Guerrero, Mariano Martínez-Vázquez, José Alberto Rivera-Chávez, Ramón Marcos Soto-Hernández, Israel Castillo-Juárez

**Affiliations:** ^1^ Laboratorio de Fitoquímica, Posgrado de Botánica, Colegio de Postgraduados, Texcoco, Mexico; ^2^Departamento de Microbiología y Parasitología, Facultad de Medicina, Universidad Nacional Autónoma de México, Ciudad de México, Mexico; ^3^Departamento de Genética Molecular, Instituto de Fisiología Celular, Universidad Nacional Autónoma de México, Ciudad de México, Mexico; ^4^Departamento de Productos Naturales, Instituto de Química, Universidad Nacional Autónoma de México, Ciudad de México, Mexico

**Keywords:** anti-virulence activity, saturated fatty acids, myristic acid, lauric acid, *Pseudomonas aeruginosa*, quorum sensing, type III secretion system, animal model

## Abstract

Blocking virulence is a promising alternative to counteract *Pseudomonas aeruginosa* infections. In this regard, the phenomenon of cell-cell communication by quorum sensing (QS) is an important anti-virulence target. In this field, fatty acids (FA) have gained notoriety for their role as autoinducers, as well as anti-virulence molecules *in vitro*, like some saturated FA (SAFA). In this study, we analyzed the anti-virulence activity of SAFA with 12 to18 carbon atoms and compared their effect with the putative autoinducer *cis*-2-decenoic acid (CDA). The effect of SAFA on six QS-regulated virulence factors and on the secretion of the exoenzyme ExoU was evaluated. In addition, a murine cutaneous infection model was used to determine their influence on the establishment and damage caused by *P. aeruginosa* PA14. Dodecanoic (lauric, C12:0) and tetradecanoic (myristic, C14:0) acids (SAFA C12-14) reduced the production of pyocyanin by 35–58% at 40 and 1,000 µM, while CDA inhibited it 62% at a 3.1 µM concentration. Moreover, the SAFA C12-14 reduced swarming by 90% without affecting biofilm formation. In contrast, CDA reduced the biofilm by 57% at 3 µM but did not affect swarming. Furthermore, lauric and myristic acids abolished ExoU secretion at 100 and 50 µM respectively, while CDA reduced it by ≈ 92% at 100 µM. Remarkably, the coadministration of myristic acid (200 and 1,000 µM) with *P. aeruginosa* PA14 induced greater damage and reduced survival of the animals up to 50%, whereas CDA to 500 µM reduced the damage without affecting the viability of the PA14 strain. Hence, our results show that SAFA C12-14 and CDA have a role in regulation of *P. aeruginosa* virulence, although their inhibition/activation molecular mechanisms are different in complex environments such as *in vivo* systems.

## Introduction

The development of anti-virulence therapies is a viable strategy to control bacterial infections, since it seeks to interfere with the production of virulence factors without directly affecting bacterial viability (Rémy et al., 2018; Scoffone et al., 2019). Quorum sensing (QS) is a cell-to-cell chemical communication phenomenon that involves the production, self-detection, and response to molecules called autoinducers (AI) (Castillo-Juárez et al., 2015). It has been proposed as one of the main anti-virulence targets because it is a global virulence regulator (LaSarre and Federle, 2013; Defoirdt, 2018).

*Pseudomonas aeruginosa* is an opportunistic pathogen that chronically colonizes the lungs of patients with cystic fibrosis (CF) and causes nosocomial outbreaks that are difficult to control due to the presence of antibiotic-resistant strains (Lister et al., 2009; Scoffone et al., 2019). Three main QS systems have been characterized in this bacterium, Las and Rhl that use AIs of the acyl-homoserine lactone type and the PQS system that uses 2-alkyl-4-quinolones (Castillo-Juárez et al., 2017). With these systems, bacteria control the acquisition of iron through siderophores (pyochelin, pyoverdine), the release of toxins (phenazines, hydrogen cyanide), the production of alginate and lipopolysaccharides, and positively regulates swarming motility and biofilm formation (Lee and Zhang, 2015).

Also, *P. aeruginosa* has various secretion systems, such as type I for alkaline protease, type II for exotoxin ExoA and elastases LasA/B (Trastoy-Pena et al., 2019), as well as the systems that inject toxins into the host epithelial cells, type VI (phospholipases D, PldA and PldB) (Jiang et al., 2014) and type III (T3SS) (ExoY, ExoT, ExoS and ExoU) that have also been proposed as anti-virulence targets (Sato and Frank, 2004; Foulkes et al., 2019). It should be noted that all these systems are regulated in an orderly manner in a complex and hierarchical network, in which transcription factors (Huang et al., 2019), regulatory RNAs (Gripenland et al., 2010), second messengers (Kalia et al., 2013) and sigma factors (Kazmierczak et al., 2005; Asfahl and Schuster, 2017) participate.

Recently, it has been reported that the regulation of *P. aeruginosa* virulence also involves fatty acids of the AI-type, belonging to the DSF family (diffusible signal factors) (Ryan et al., 2015; Zhou et al., 2017). Two main regulatory mechanisms have been described, the first of which has been related to inter-genera communication, since the system contains the PA1396 sensor kinase that only perceives DSF produced by other bacteria (Ryan et al., 2008). In the second system, the bacterium synthesizes *cis*-2-decenoic acid (CDA, C10:1) through a putative enoyl-coenzyme A hydratase/isomerase called DspI (*Dispersion inducer*) (Amari et al., 2013). Although the receptor for CDA has not been identified, it has been shown to positively regulate pyoverdine production and swarming, in addition to inducing biofilm dispersion and promoting systemic dispersal in lung infections in mice (Liu et al., 2018). During the evaluation of QS systems in monocultures *in vitro*, the effect of host and microenvironmental elements is generally not considered, neither the polymicrobial nature of the infections (Twomey et al., 2012; Welsh and Blackwell, 2016; Tan et al., 2017; Mukherjee and Bassler, 2019). In some microenvironments such as the lung tissue of patients with CF, the presence of saturated fatty acids (SAFA) has been reported, mainly myristic and palmitic acid (Son et al., 2007) and also monounsaturated fatty acids such as DSF produced by bacteria associated with the infection such as *Burkholderia cenocepacia* and *Stenotrophomonas maltophilia*, which alter the virulence of *P. aeruginosa* (Boon et al., 2008; Deng et al., 2013).

Fatty acids (FA) are widely distributed molecules in nature that have structural functions, are used as carbon sources and also have bactericidal properties (Sun et al., 2012; Calder, 2015). Moreover, at sublethal concentrations, some FA act as signal molecules in the regulation of bacterial virulence (Cortes-López et al., 2020). Furthermore, lauric (C12:0), myristic (C14:0), palmitic (C16:0) and stearic (C18:0) acids have been reported to reduce some virulence factors of *Proteus mirabilis* (Liaw et al., 2004), *Chromobacterium violaceum* (Pérez-López et al., 2017), *Vibrio* sp (Widmer et al., 2007; Santhakumari et al., 2016; Santhakumari et al., 2017) and *P. aeruginosa* (Inoue et al., 2008; Abd-Alla and Bashandy, 2012). Although the exact mechanism of action of these molecules is not known, it has been suggested that it is due to interference with QS systems (Widmer et al., 2007; Pérez-López et al., 2017).

Thus, we do not yet understand the role of FA as environmental signals and their participation in the complex network of virulence regulation, as well as their possible interference in the efficacy of anti-virulence therapies. This research aimed to evaluate the anti-virulence activity of SAFA C12-18, as well as to compare their effect with the putative autoinducer CDA in a cutaneous infection model in mice. Our results shed light on the role of SAFA in the host environment and how they influence the pathogenicity of *P. aeruginosa*.

## Materials and Methods

### Bacterial Strains and Growth Conditions

Bacterial strains of *P. aeruginosa* used in this study are listed in **Supplementary Table 1**. We used two isogenic derivates of PA14 WT strain, for QS (Δ*lasR*/Δ*rhlR*) and T3SS (Δ*pscC*). They were kept in 10% glycerol at -70 °C. The pre-cultures were grown in LB broth at 37 °C with 250 rpm shaking overnight and then adjusted to an O.D. _620 nm_ of 0.05 with the different treatments. For the assays of alkaline protease, elastase, pyocyanin and hemolysis the cultures were incubated for 16 h.

### Fatty Acids

SAFA (EC10A-1KT Supelco: lauric, myristic, palmitic and stearic) and *cis*-2-decenoic acid (Sigma) were dissolved in dimethyl sulfoxide (DMSO) at 40–1,000 µM for SAFA and 3.1–500 µM for CDA.

### Bacterial Growth Analysis

Bacterial cultures were grown overnight in LB broth plates with 96 wells. They were adjusted to an O.D. _620 nm_ of 0.05 with or without lauric or myristic acids using 40, 200, and 1,000 µM of each one. The cultures were incubated at 37 °C with 250 rpm shaking. Measurements were taken every two hours for 24 h.

### Pyocyanin Assay

Pyocyanin was determined as Essar et al. (1990) with modifications. Bacterial cultures were centrifuged at 10,000 rpm for 3 min and 800 µl of supernatants were collected. The supernatants were vortexed with 420 µl of chloroform for 2 min. The samples were centrifuged for 8 min at 10,000 rpm. The organic phase was collected (chloroform) and it was added to 800 µl of 0.2 N HCl. The samples were vortexed for 1 min. Then 650 µl of the aqueous phase were taken and 650 µl of distilled water were added. The pyocyanin was determined at 520 nm. The data were normalized with respect to cellular density.

### Alkaline Protease Assay

The inoculum was centrifuged (1–2 ml) at 13,000 rpm for 1 min. The supernatants were taken and filtered with 0.45 µm filters. 5 mg of hide remazol brilliant blue R (Sigma) were added in 1.5 ml micro-centrifuge tubes and 875 µl of reaction buffer (20 mM Tris-HCl pH 8.0, 1 mM CaCl_2_) and 125 µl of supernatant were added. The tubes were incubated at 37 °C with 200 rpm shaking for 1 h. The mixture was centrifuged at 13,000 rpm for 5 min and the supernatant was taken. The absorbance was determined at 595 nm. The data were normalized with respect to cellular density.

### Elastase Assay

The cultures were centrifuged at 13,000 rpm for 1.5 min and the supernatants were taken, then they were diluted 1:10 with elastase buffer. Fifty µl of diluted supernatant were added in a vial with 5–8 mg of elastin red Congo. The vials were incubated at 37 °C with agitation at 200 rpm for 2 h. The absorbance was determined at 495 nm. The data were normalized with respect to cellular density.

### Motility Assay

M9 agar was used for motility assays (Kollaran et al., 2019). Three ml per well were added in a plate with six wells, with or without SAFA or CDA. Five µl of inoculum were loaded at the center of each well. The plates were incubated at 37 °C for 24 h under humidity conditions. The ImageJ^®^ program was used to calculate the swarming area.

### Biofilm Assay

Biofilms were quantified as previously reported (O’Toole, 2010). A PA14 culture was adjusted to an O.D. _620 nm_ of 0.08 and 200 µl per well were deposited onto a plate of 96 wells. The plate was incubated for 24 h at 37 °C with shaking at 250 rpm. The culture was discarded, and the plate was washed with distilled water three times and dried at 40 °C for 20 min. Two hundred µl of 0.1% violet crystal were added per well and the plate was washed three times to eliminate the excess of the colorant. The plate was dried at environmental temperature for one hour and the adhered colorant was suspended with 200 µl of ethanol 80% per well. The absorbance was determined at 570 nm. The data were normalized with respect to cellular density.

### Hemolysis Assay

The hemolysis assay was performed as previously reported (Rowe and Welch, 1994). Square plates with 5% of defibrinated blood (v/v) were prepared with wells of 3.8 mm. The inoculum was centrifuged at 10,000 rpm for 3 min. Five µl were taken and loaded onto the wells of the plates that were incubated at 37 °C for 16 h. The halo diameter was measured.

### Type III Secretion Assay

The type III protein secretion assay was performed as previously reported (Soto-Aceves et al., 2019). Secreted proteins were precipitated from the supernatant using trichloroacetic acid, resuspended in SDS-loading buffer according to the O.D. _600 nm_ of each culture and separated in a 15% SDS-PAGE. Proteins were detected by Western blot analysis using anti-ExoU polyclonal antibodies and a chemiluminescent detection system (Millipore). Western blot images were quantified using the Image Studio Lite software.

### Cutaneous Infection (Abscess) Model

CD1 mice of six to eight weeks were obtained from the Facultad de Estudios Superiores, Iztacala, UNAM. Animals were kept in standard conditions (23 °C ± 2 °C) with a 12 h light-dark cycle and allowed free access to food and water.

Fresh cultures of PA14 were adjusted to an O.D. _600 nm_ of 0.08 (≈10^7^ CFU/60 µl). They were centrifuged at 13,000 rpm for 2 min and washed with phosphate buffered saline (PBS); this process was repeated three times. Subsequently, they were resuspended in 1,500 µl of PBS with or without the different treatments previously dissolved in DMSO. It should be noted that DMSO was also added to the untreated controls in an amount equivalent (final concentration ≤ 2.5%) to that of the treatment groups.

The inoculation was performed as previously reported (Pletzer et al., 2017). Mice were shaved with a shaver and chemical depilatories on the right flank. They were anesthetized with an intraperitoneal injection of sodium pentobarbital, then 60 µL of the inoculum with the different treatments were injected into the subcutaneous space. The infection process was monitored every 24 h for 4 days. The abscess area was measured the first day, and necrotic areas were measured for 4 days. After this period, animals were sacrificed, and the necrotic areas and livers were resuspended and homogenized in 3 ml of PBS. Serial dilutions were performed in LB plates to determinate *log* CFU/g.

### Statistical Analysis

*In vitro* experiments were carried out at least in triplicate while the cutaneous infection model was done at least twice. Statistical analysis for *in vitro* assays was evaluated by using a Student’s t distribution test (*α < 0.05, **α < 0.01). The non-parametric test U-Mann-Whitney (*α < 0.05, **α < 0.01) was used for the cutaneous infection model. All the analysis was performed with SPSS Statistics Version 25 statistical package.

### Ethical Declaration

The indications of regulation for use and care of animals destined for research at the Hospital Infantil de México-Federico Gómez were always followed (HIM2018-002).

## Results

### SAFA Reduce Some QS-Regulated Virulence Factors

**Figure 1:**
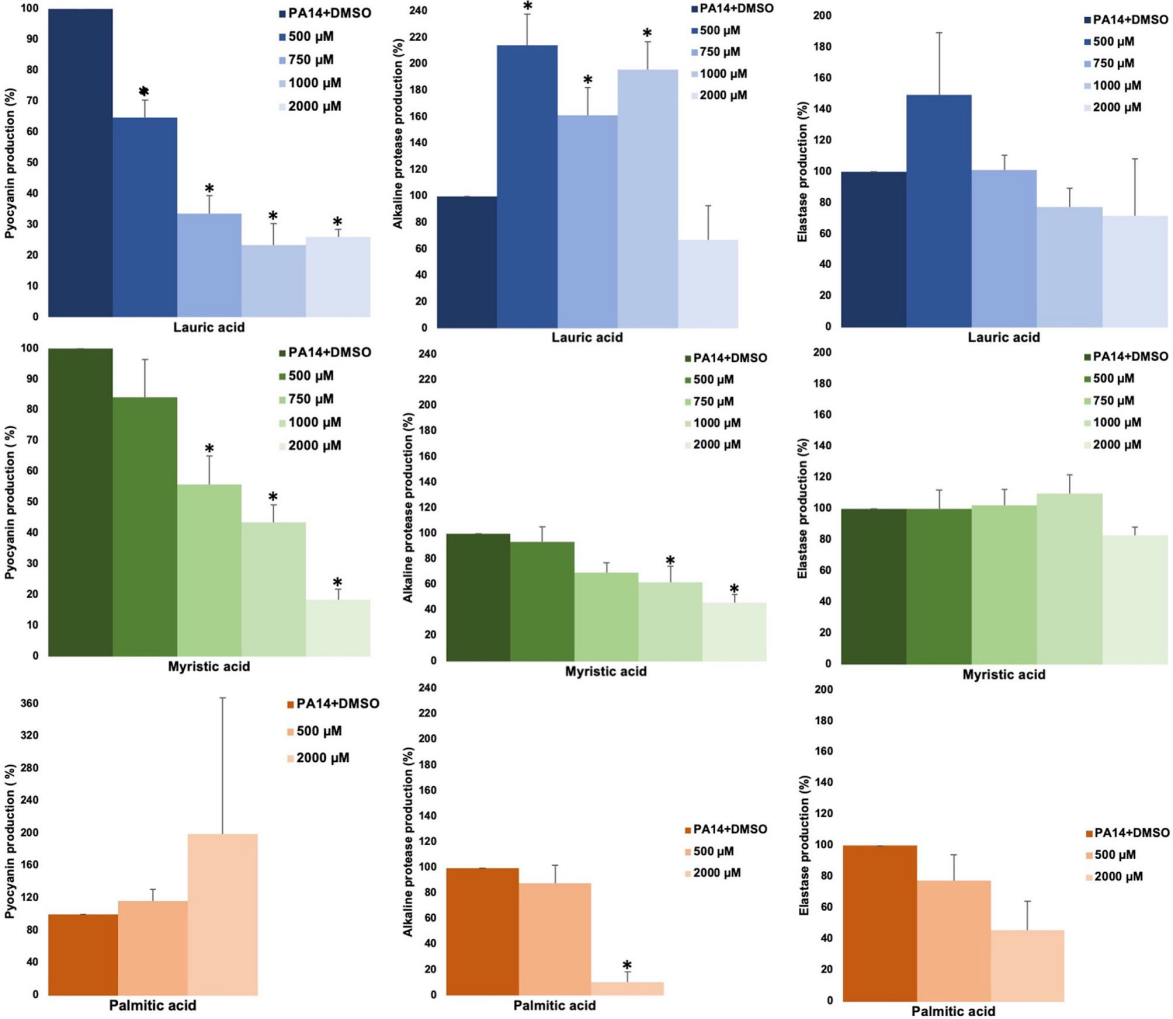
Effect of SAFA C12-16 on virulence factors regulated by QS in *P. aeruginosa*. The results shown are the means of three independent experiments and standard error (*α ≤ 0.05). DMSO was added to the untreated groups in an equivalent proportion (≤ 2.5%) to that of the treatment groups.

The effect of SAFA C12-18 [lauric (C12:0), myristic (C14:0), palmitic (C16:0), and stearic (C18:0) acids] on the production of virulence factors regulated by QS (pyocyanin, elastase, alkaline protease, and hemolysis) in *P. aeruginosa* was analyzed. Lauric and myristic acids inhibited pyocyanin production in a dose-response way by about 50–70% at concentrations from 500 to 2,000 µM, without affecting growth (**Figure 1**, **Supplemental Figure 1**). Lauric acid increased alkaline protease activity with intermediate concentrations (500–100 µM), while myristic acid (1,000 and 2,000 µM) and palmitic acid (2000 µM) inhibited it by 50–90% (**Figure 1**). Stearic acid did not show activity on the evaluated virulence factors (data not shown) and none of the SAFA inhibited hemolytic activity (**Supplemental Figure 2**).

### CDA Inhibits Pyocyanin Production

**Figure 2:**
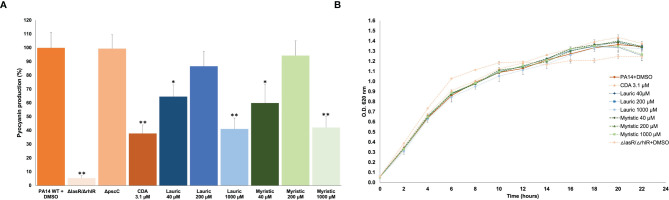
Effect of SAFA C12-14 and CDA on pyocyanin production **(A)** and growth **(B)** of *P. aeruginosa*. Δ*lasR*/Δ*rhlR*, QS-mutant strain; CDA, *cis*-2-decenoic acid. Student’s t distribution test (*α ≤ 0.05, **α ≤ 0.01). The results shown are the mean and standard error of four independent assays with three replicates each one.

The effect of SAFA C12-14 and CDA on pyocyanin production was compared. In this test, lauric and myristic acids inhibited pigment production (in a non-dose-response relationship) from 35.4 to 58.8%, at 40 and 1,000 µM concentrations. In contrast to what was expected, CDA showed a 62% inhibition at a 3.1 µM concentration (*α* ≤ 0.05) (**Figure 2A**). In the Δ*lasR*/Δ*rhlR* mutant strain used as a positive control pyocyanin production was reduced by 94.6% (**Figure 2A**). It should be noted that this effect occurred without altering bacterial growth (**Figure 2B**).

### Lauric and Myristic Acids Inhibit Swarming but Not Biofilm Formation

**Figure 3:**
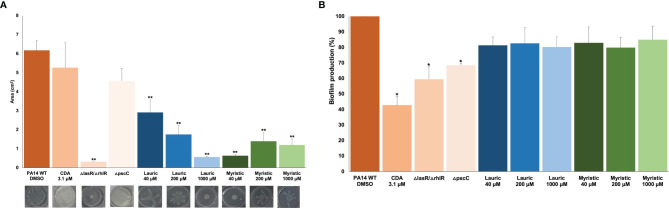
Effect of lauric acid, myristic acid, and CDA on swarming **(A)** and biofilm production **(B)**. CDA, *cis*-2-decenoic acid; Δ*lasR*/Δ*rhlR*, QS mutant strain; Δ*pscC*, T3SS mutant. Student’s t test (*α < 0.05, **α < 0.01). The results shown are the means of three repetitions and standard error. DMSO was added to the untreated groups in an equivalent proportion (≤ 2.5%) to that of the treatment groups.

The main anti-virulence effect of SAFA C12-14 was on swarming. Lauric acid showed a dose-response effect with a maximum inhibition of 91% at 1,000 µM, while for myristic acid it was 90% at 40 µM (**Figure 3A**). This reduction in swarming was similar to the one observed in the Δl*asR*/Δ*rhlR* mutant strain (95%), used as a positive control (**Figure 3A**). On the other hand, none of these fatty acids showed a significant effect on biofilm formation, although it was reduced by 41% in the Δ*lasR*/Δ*rhlR* mutant (*α* ≤ 0.05) and 32% in the *ΔpscC* mutant (*α* ≤ 0.05) (**Figure 3B**).

Conversely, CDA reduced biofilm formation by 57% (**Figure 3B**) but did not significantly affect swarming at a concentration of 3.1 µM (**Figure 3A**). Similarly, swarming was not affected in the Δ*pscC* mutant strain (**Figure 3A**), but biofilm production was reduced by 30% (**Figure 3B**).

### SAFA Inhibit ExoU Secretion

**Figure 4:**
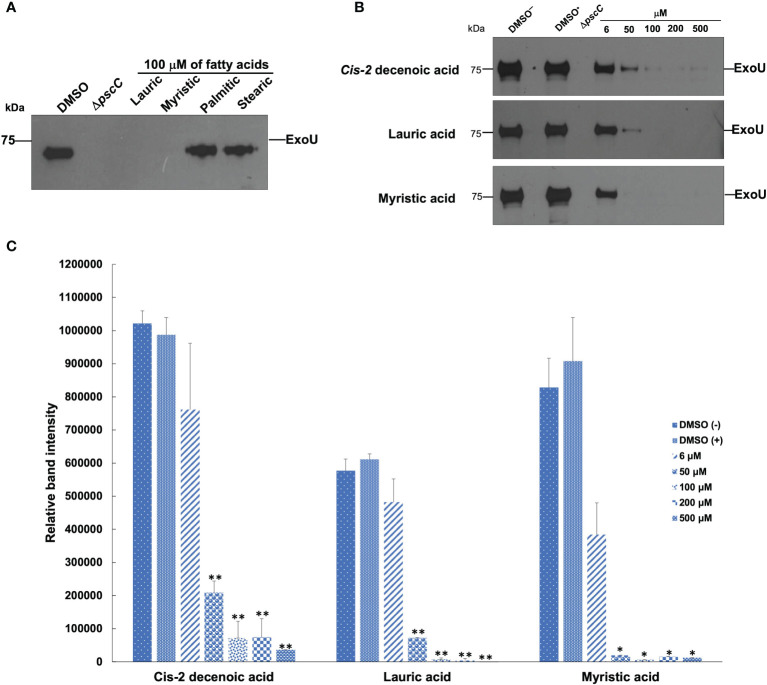
Effect of SAFA and CDA on ExoU secretion. SAFA C12-18 **(A)**. CDA, lauric, and myristic acids in a concentration gradient **(B)**. The intensities of the bands were quantified and are shown in **(C)**. Values represent the mean ± standard error of two independent experiments and levels are expressed relative to the wild-type strain (*α < 0.05, **α < 0.01; Student’s t test).

Lauric and myristic acids were the most effective at inhibiting effector secretion since the presence of ExoU was not detected in the supernatants at 100 and 50 µM concentration, respectively, (**Figures 4A–C**). This effect is like that exhibited by the *ΔpscC* mutant strain, in which ExoU is not secreted (**Figure 4**). However, with the longer chain SAFA, such as palmitic and stearic acids, the activity was not markedly reduced (**Figure 4A**). Interestingly, CDA at 100 and 200 µM concentrations reduced effector protein secretion by 92%, reaching a maximum inhibition of 96% at 500 µM (**Figures 4B, C**). Under these experimental conditions, there was no effect on the growth of the bacteria (**Supplemental Figure 3**).

### Myristic Acids Promote *P. aeruginosa* Damage and Dispersal *In Vivo*

Intramuscular inoculation of PA14 (10^7^ CFU) generated an abscess of 171.9 mm^2^ in 24 h and a maximum necrotic area of 15.6 mm^2^ in 48 h (**Supplementary Figures 4A** and **B**). Additionally, at 96 h (maximum experimentation time) an establishment of *log* 7 CFU/g was registered in the infection zone, as well as dispersion of bacteria to the liver (*log* 0.7 CFU/g) and a survival of 71% (**Table 1**).

**Table 1:** Influence of SAFA C12-14 on the pathogenicity of *P. aeruginosa* in the cutaneous infection model.

Treatments	Abscess area (mm^2^, mean ± S.E.)	Necrotic area (mm^2^, mean ± S.E.)	Inoculation area (*log* CFU/g)	Liver (*log* CFU/g)	Survival (%)
PA14 wt	171.9 ± 1.14	15.6 ± 5.6	7.0 ± 1.3	0.7 ± 0.3	71.4
Δ*lasR*/Δ*rhlR*	120.4 ± 25.7*	7.3 ± 3.6	4.5 ± 1.2	0.6 ± 0.3	81
Δ*pscC*	42.7 ± 15.1**	0 ± 0**	0 ± 0**	0.3 ± 0.3	100
PA14 wt + lauric acid (µM)					
40	182.2 ± 9.3	25.5 ± 7.9	7.8 ± 0.8	2.2 ± 0.9	50
200	149.3 ± 17.5	34.6 ± 10.1*	7.3 ± 0.9	1.7 ± 0.7	90
1,000	201.3 ± 17.7	40.6 ± 13.1	7.0 ± 1.8	0.3 ± 0.3	50
PA14 wt + myristic acid (µM)					
40	191.6 ± 39.8	28.4 ± 7.9	7.5 ± 0.4	1.6 ± 1.0	50
200	178.9 ± 22.5	36.7 ± 6.1*	8.0 ± 0.3	3.3 ± 0.6**	70
1,000	230.4 ± 21.2*	32.6 ± 5.7*	8.5 ± 0.1	2.9 ± 0.6**	60

The abscess area was determined at 24 h and the necrotic area at 48 h. Injured tissues and liver were harvested 96 h after infection, homogenized, and the number of CFU/g of tissue was calculated. Experiments were done on five mice per group and were performed at least twice. Values in bold denote statistical significance with the PA14wt treated group. (*α < 0.05, **α < 0.01; Mann-Whitney test). ΔlasR/ΔrhlR, QS mutant. ΔpscC, T3SS mutant. DMSO was also added to the untreated controls in an amount equivalent (final concentration ≤ 2.5%) to that of the treatment groups.

In the groups inoculated with the mutant strains and used as negative controls, there was less damage inflicted to the tissues. In the Δ*lasR*/Δ*rhlR* mutant strain, the abscess formation was 120.4 mm^2^ (*α* ≤ 0.05) and a necrotic zone of 7.3 mm^2^; with an establishment in the infection area of *log* 4.5 CFU/g, a spread to the liver of *log* 0.6 CFU/g and a survival of 81% (**Table 1**). Besides, the Δ*pscC* mutant showed the lowest virulence of the entire study since it only generated an abscess of 42.7 mm^2^ (*α* < 0.01) and no necrosis (*α* < 0.01). Moreover, bacteria were not present in the inoculation zone (*α* < 0.01), and although a low number of bacteria was detected in the liver (*log* 0.3 CFU/g), a 100% survival of the animals was maintained (**Table 1**).

Remarkably, and in contrast to what was expected, in the groups in which SAFA C12-C14 were co-administered with bacteria, there was a tendency to induce systemic dispersion and damage caused by PA14 (**Table 1**, **Supplementary Figures 5–8**). In the group co-administered with lauric acid, a greater area of necrosis was induced with 200 and 1000 μM (**Table 1**, **Supplementary Figures 4A, 5** and **6**), in addition, survival was reduced by 50% at the highest dose.

However, the effect was more consistent with myristic acid, which with 200 μM induced a necrotic area of 36 mm^2^ (α < 0.05) and a systemic dispersion of *log* 3.3 CFU/g (α < 0.01), which was greater to that observed for PA14 without treatment. While at 1,000 μM it induced an abscess of 230 mm^2^ (α < 0.01), a necrotic area of 32 mm^2^ (α < 0.05) and a systemic dispersion of *log* 2.9 CFU/g (α < 0.01) (**Table 1**, **Supplementary Figures 4B, 7** and **8**). It should be noted that the administration of SAFA C12-14 without the bacteria does not induce inflammation or necrosis (**Supplemental Figure 9**).

Thus, to distinguish in which of the two systems (QS or T3SS) lauric and myristic acids were acting, their effect on the mutant strains *in vivo* was evaluated. However, none of them stimulated the pathogenicity in the cutaneous infection model (**Table 2** **Supplementary Material** and **Supplementary Figures 10, 11**), which suggests that the induction of virulence in PA14 (**Table 1**) requires both systems to be active.

**Table 2:** Influence of CDA on the pathogenicity of *P. aeruginosa* in the cutaneous infection model.

Treatments	Abscess area (mm^2^, mean ± S.E.)	Necrotic area (mm^2^, mean ± S.E.)	Inoculation area (*log* CFU/g)	Liver (*log* CFU/g)	Survival (%)
PA14 wt	274.5 ± 40.7	20.4 ± 6.5	5.2 ± 1	1.5 ± 1	57.9
PA14 wt + CDA (µM)					
3.1	260.2 ± 22.1	31.4 ± 10.7	5.1 ± 1.1	0.6 ± 0.6	72.2
500	82.4 ± 24.2**	7.1 ± 3.5*	1.13 ± 0.73**	0.9 ± 0.64	88.9

The abscess area was determined at 24 h and the necrotic area at 48 h. Injured tissues and liver were harvested 96 h after infection, homogenized, and the number of CFU/g of tissue was calculated. The experiment was carried out three times with five to eight mice per group. Values in bold denote statistical significance with the PA14wt treated group. (*α < 0.05, **α < 0.01; Mann-Whitney test). ΔlasR/ΔrhlR, QS mutant. ΔpscC, T3SS mutant. DMSO was also added to the untreated controls in an amount equivalent (final concentration ≤ 2.5%) to that of the treatment groups.

### CDA Reduces Damage Caused by PA14

In the *in vivo* infection assay, PA14 (10^7^ CFU) induced an abscess of 274.5 mm^2^ (24 h) and a maximum necrotic area of 20.4 mm^2^ (48 h); as well as an establishment in the infection zone of *log* 5.2 CFU/g and a survival of 57.9% (**Table 2**). However, in the groups in which PA14 was co-administered with CDA, there was less damage produced. Mainly, at the concentration of 500 µM, the area of abscess (82.4 mm^2^, α < 0.01) and necrosis (7.1 mm^2^, α < 0.05) were reduced, as well as the establishment of infection (*log* 1.1 CFU/g, α < 0.05) (**Table 2**, **Supplementary Figures 4C** and **12**). Furthermore, there was a liver dispersion of *log* 0.9 CFU/g (not significant) and a survival of 88.9% (**Table 2**).

## Discussion

Anti-virulence strategies against *P. aeruginosa* have mainly focused on the inhibition of the three QS systems (Las, Rhl, and PQS) that are best understood (Castillo-Juárez et al., 2017). However, the role of FA as signaling molecules involved in the regulation of virulence has gained relevance in recent years (Sun et al., 2012; Cortes-López et al., 2020; Kumar et al., 2020), although its role *in vivo* is still little explored. It has been suggested that during the development of chronic infections such as in CF patients, the DSF of other bacteria associated with the infection of *P. aeruginosa* influence the efficacy of treatments (Twomey et al., 2012). Also, the consumption of SAFA in the diet can limit the development of infections (Pérez-López et al., 2017). Thus, to elucidate the regulatory role of SAFA C12-18 on some virulence factors, their effect was compared with that of CDA, which is a putative autoinducer produced by *P. aeruginosa* and a recent member of the DSF family. In this regard, DSF are *cis*-monounsaturated FA, with chains of 10 to 16 carbon atoms, mostly methylated (Cortes-López et al., 2020). In the case of CDA, the DspI synthase forms the double bond in the FA precursor (Amari et al., 2013).

We have identified an inhibitory effect in the production of pyocyanin by lauric (C12:0) and myristic (C14:0) acids, but also unexpectedly with CDA (**Figure 2**), which is considered a QS signal molecule (Liu et al., 2018). Pyocyanin is an important virulence factor since it causes oxidative stress in host cells, increases the survival of bacteria under anaerobic conditions, and induces resistance to toxic metals (Muller and Merrett, 2014). Our results agree with previous studies showing that myristic acid inhibits pyocyanin production (Abd-Alla and Bashandy, 2012). However, except for myristic acid, which also inhibited protease activity (**Figure 1**), SAFA C12-18 did not inhibit other QS-regulated phenotypes such as elastase, hemolytic and proteolytic activities (**Figure 1** and **Supplementary Figure 2**). Therefore, other independent pathways of QS regulation may be involved in the inhibition of pyocyanin by FA, as is the case with several specific anti-pyocyanin metabolites (Miller et al., 2015). Moreover, unexpectedly the low concentration of 40 µM of lauric and myristic acids significantly inhibited the production of pyocyanin while a higher concentration of 200 µM, highlighting the complexity of the regulatory network that control the expression of virulence factors in *P. aeruginosa*.

Swarming motility and biofilm formation are multicellular behaviors positively regulated by the Las and Rhl systems (Chrzanowski et al., 2012). In this study, we found opposite effects of SAFA and CDA on these phenotypes. Lauric and myristic acids strongly inhibited swarming but did not clearly reduce biofilm formation; while CDA strongly reduced biofilm but did not affect swarming (**Figure 3**). This is in agreement with previous studies that report the reduction of swarming by myristic acid in PAO1 (Inoue et al., 2008) and the biofilm dispersing capacity of CDA (Davies and Marques, 2009; Amari et al., 2013; Liu et al., 2018).

Furthermore, although the positive regulation of the expression of the T3SS by QS in *P. aeruginosa* is not yet clear (Hogardt et al., 2004; Trastoy-Pena et al., 2019), the exoenzyme ExoU is recognized as the most toxic effector protein of those secreted by this system (Sato and Frank, 2004). ExoU is a phospholipase A_2_ that causes rapid cell lysis of the membranes of epithelial cells, macrophages, and neutrophils, generating an immunosuppressive effect that favors the development of secondary infections (Sato et al., 2003; Foulkes et al., 2019). In this work, we report an inhibitory activity of ExoU secretion by lauric and myristic acids, as well as by CDA (**Figure 4**). In this regard, it has been reported that the external addition of non-esterified FA (oleate, myristate and palmitate) represses the expression of *hilD*, which encodes the transcriptional activator of the structural genes of the SPI1-T3SS in *Salmonella enterica* serovar Typhimurium (Golubeva et al., 2016). Likewise, 100 µM of *cis*-2-dodecenoic acid (BDSF, C12:1 produced by *B. cenocepacia*) reduces T3SS gene expression and ExoS effector secretion in *P. aeruginosa* (Deng et al., 2013). Hence, the inhibition of ExoU (as well as other prokaryotic lipases) by FA is important for the development of anti-virulence therapies (Bialecka-Florjanczyk et al., 2018; Foulkes et al., 2019).

The *P. aeruginosa* Las and Rhl QS systems have been shown to be determinant in causing damage and death in several animal models of infection, including murine and insects (Castillo-Juárez et al., 2015). Similarly, the T3SS is necessary to generate damage in mammalian hosts, although it does not participate in the induction of death of the nematode *Caenorhabditis elegans* (Feinbaum et al., 2012). However, it was reported that DspI is necessary to induce the death of *C. elegans*, and the deletion of the *dspI* gene reduced the dispersion of the biofilm, the production of pyoverdine, the swarming and the systemic dispersal in a lung infection model in mice (Liu et al., 2018).

Here, we used a model of cutaneous infection (abscess), and in contrast to the *in vitro* anti-virulence results, lauric and myristic acids favored the damage and establishment of the bacteria in mice (**Table 1**). Even more interesting, CDA at concentrations of 3 µM did not stimulate the damage and strongly reduced it at 500 µM without affecting the growth of bacteria (**Table 2**). In this regard, a similar effect was reported with BDSF (C12:1), which increased the survival of zebrafish infected with PA14 by 50% at 24 h post-infection (Deng et al., 2013). Thus, although these are promising results, more *in vivo* studies related to CDA and its possible usefulness in anti-virulence strategies are necessary.

The opposite results obtained between *in vitro* studies and the murine model indicate how important the microenvironment is in the development of infection. Although there are still very few studies in this regard, it has been shown that some environmental and host factors interfere in the regulation of QS and secretion systems, modulating the outcome of bacterial invasion (Lee et al., 2013; Welsh and Blackwell, 2016; Mukherjee and Bassler, 2019). For example the activity of ExoU requires eukaryotic cofactors so it is achieved only in the presence of mammalian cell lysates, specifically with ubiquitin or ubiquitinated proteins (Tyson and Hauser, 2013). Also, in CF patients, the main source of FA is lung surfactant, made up of 90% lipids, of which 80% is phosphatidylcholine (Son et al., 2007), which is degraded by lipases releasing mainly myristic (10–20%) and palmitic (50–60%) acids. Thus, SAFA under stress conditions (*in vivo*) may act as signal molecules and participate in signaling pathways other than those registered under ideal conditions *in vitro*.

*P. aeruginosa* has a high plasticity to adapt to various environments and uses various carbon sources (Kang et al., 2010). Also, it is capable of degrading FA of different aliphatic chain sizes such as short, medium (C4-C10) and long (≥ C12) (Stover et al., 2000). However, although the degradation of long-chain FA (LCFA) can be carried out by other β-oxidation pathways (*fadAB1* and *fadAB4* operons) (Son et al., 2007), the only one that is related to the adaptation of bacteria to the stationary phase is the one carried out by *fadBA5* (Kang et al., 2008). In *P. aeruginosa*, several transcriptional factors (TF) and sigma factors have been described that regulate QS and the T3SS in response to microenvironmental changes (Kazmierczak et al., 2005; Asfahl and Schuster, 2017). Recently, twenty TF were identified that bind to 1,200 genes that regulate in an overly complex network the expression of QS and secretion systems in this bacterium (Huang et al., 2019).

PsrA is a global transcriptional regulator and main repressor of the Fad pathway of FA degradation (Kojic et al., 2005; Son et al., 2007; Kang et al., 2008), and it also controls the expression of virulence (Kojic et al., 2002; Kang et al., 2009). PsrA represses the *fadBA5* operon, as well as *PA0506* which encodes the FadE protein, a key enzyme in the initiation of the β-oxidation cycle (Kojic et al., 2005; Kang et al., 2008; Wells et al., 2017). The repression of FA degradation generates the accumulation of acyl-CoA that favors the synthesis of PQS (Wells et al., 2017).

**Figure 5:**
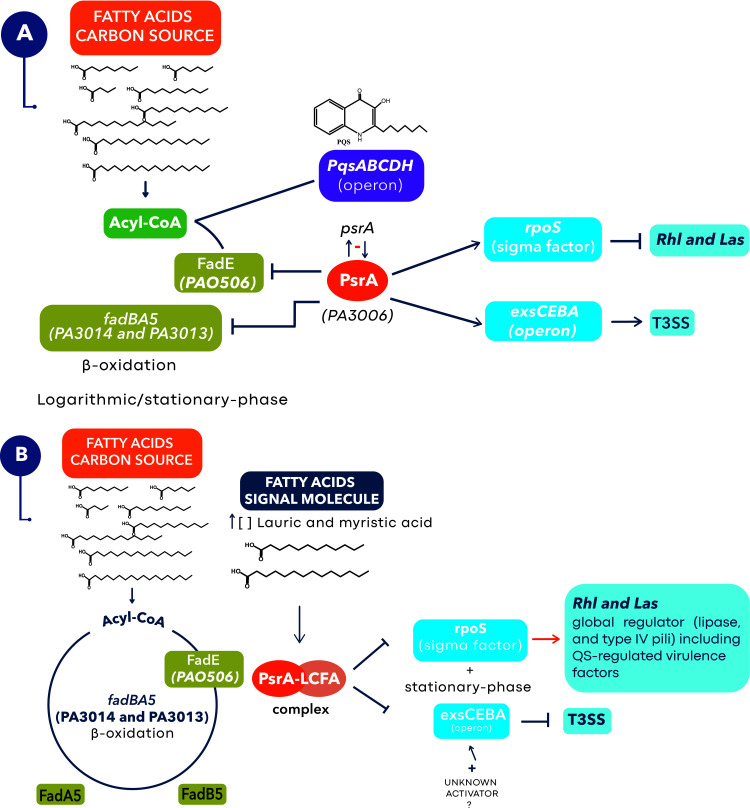
Proposed model for the role of lauric and myristic acids with PsrA at a higher level of regulation of pathogenicity in *P. aeruginosa*. **(A)** In late logarithmic growth phase PsrA binds to the promoters of *fadBA5* and *PA056*, repressing β-oxidation through the fadBA5 pathway (Kojic et al., 2005; Son et al., 2007; Kang et al., 2008) and allowing the synthesis of PQS by blocking the expression of FadE (Wells et al., 2017). Similarly, PsrA maintains a negative self-regulation over its *psrA* promoter region (Kojic et al., 2002), but positively regulates *rpoS* transcription which prevents early activation of Rhl and Las QS systems (Pearson, 2002; Schuster et al., 2004). In this scenario, PsrA maintains a positive regulation over the *exsCEBA* operon needed to transcribe the T3SS genes (Kang et al., 2009) and for the secretion of ExoU, a lipase that will favor an increase in the concentration of lauric and myristic acids in the microenvironment of the host cell saponifiable lipids (Foulkes et al., 2019). **(B)** We suggest that an increase in the concentration of lauric and myristic acids (signal molecules) in the stationary phase will favor the formation of a PsrA-LCFA complex that removes the repression of the *fadBA5*, *PA056*, and *rpoS* promoter regions (Kang et al., 2008; Kang et al., 2009; Wells et al., 2017), activating β-oxidation through the fadBA5 pathway and the production of virulence factors regulated by the Rhl and Las QS systems (Pearson, 2002; Schuster et al., 2004). Meanwhile the PsrA-LCFA complex prevents the binding of PsrA with the promoter region of *exsCEBA* stopping the transcription of the T3SS genes (Kang et al., 2009). At this stage, some microenvironment or host factors (e.g., an unknown activator) could reactivate expression of the T3SS.

In *Pseudomonas* the perception of environmental changes in the stationary phase is made by the sigma factor RpoS (Potvin and Levesque, 2008). RpoS has been identified as a negative regulator of the Rhl and Las systems preventing their early activation (Schuster et al., 2004). PsrA positively regulates *rpoS* transcription, so in stationary growth phase (or late logarithmic) its expression is active and therefore that of several virulence factors is repressed (Schuster et al., 2004). Also, PsrA negatively self-regulates by binding to its *psrA* promoter region, and it also controls RpoS concentration by binding to the *rpoS* promoter region (Kojic et al., 2002) (**Figure 5A**).

Interestingly, PsrA has a function as an LCFA sensor, with which it forms the PsrA-LCFA complex that derepresses *fadBA5* and *PA0506*, activating the β-oxidation and stopping the production of PQS (Kang et al., 2008; Kang et al., 2009; Wells et al., 2017). Specifically, the formation of the PsrA-LCFA complex with 50 µM of lauric, myristic, palmitic, and oleic (C18:1Δ^9^) acids was identified through an electrophoretic mobility shift assay (EMSA) (Kang et al., 2008; Wells et al., 2017). Furthermore, the expression of *fadBA5* occurs only in the middle exponential phase, which is related to the presence of LCFA in the medium (Kang et al., 2009). In contrast, the presence of glucose and short-chain FA as the only carbon sources strongly represses the expression of *fadBA5* (Kang et al., 2009). Additionally, the interaction of LCFA with PsrA was confirmed with the crystallization of the protein and by directed mutagenesis of the hydrophobic channel, which results in no binding of LCFA and hence, no activation of *rpoS* and *exsC* (Kang et al., 2009).

Likewise, in patients with CF, the joint expression of *psrA* and *fadBA5* has been observed, which suggests that bacteria detect free FA in the lung so that PsrA no longer represses the expression of *fadAB5* (Son et al., 2007). Similarly, *fadBA5* is necessary for the degradation of phosphatidylcholine and FA *in vitro* (Son et al., 2007). Finally, PsrA also positively regulates the *exsCEBA* operon by binding to the *exsC* promoter region that transcribes the genes needed for expression of the T3SS (Kang et al., 2009). So, with this information and the results obtained, we propose an integrated model for the role of lauric and myristic acids in the regulation of virulence (**Figure 5**).

The co-administration of PA14 with SAFA C12-14 (≥ 200 µM) induces the formation of the PsrA-LCFA complex that favors the activation of Rhl and Las systems, as well as other virulence factors such as the T6SS, lipases, and type IV pili (**Figure 5B**). However, the formation of the complex inactivates the expression of the T3SS and therefore the secretion of ExoU, consistent with the results in which SAFAC12-18 reduces its secretion (**Figures 4A, B**). However, indirect evidence suggests that the T3SS remains active and that it participates in the damage induced by SAFA C12-14. i) inactivation of ExoU secretion reduces damage, as demonstrated with the Δ*pscC* mutant strain that cannot assemble the T3SS nano-syringe (**Table 1**). ii) the SAFA C12-14 did not induce virulence in the mutant strains Δ*lasR*/Δ*rhlR* and Δ*pscC*, which suggests that both systems (QSS and T3SS) are necessary for pathogenicity to be induced (**Supplementary Table 2**). iii) in the mouse cutaneous infection model, the ExoU effector is necessary to induce damage (Berube et al., 2017).

We suggest that some unidentified host or microenvironmental factor could be reactivating the T3SS *in vivo*. In this regard, it has been identified that ExsA is one of the relevant TF in QS, T3SS and T6SS regulation in *P. aeruginosa* (Huang et al., 2019). It is also one of the main regulators of ExoU expression and it is activated in response to environmental signals, such as the decrease in calcium concentration and the interaction with the host tissue (Ha et al., 2004; Hogardt et al., 2004). The global understanding of these mechanisms will help to develop more successful anti-virulence combination therapies, in which the administration of lipase inhibitors together with inhibitors of secretion systems or QS, can increase their efficiency.

Finally, although the receptor protein for CDA is unknown, it has been identified that it plays an important role in the regulation of 666 genes involved in 15 cellular processes, including motility, swarming, virulence, and persistence among others (Rahmani-Badi et al., 2015). Thus, in comparison with the previously reported effect of BDSF, which reduces ExoS secretion and pathogenicity in an animal model by a mechanism independent of PsrA (Deng et al., 2013), we suggest that CDA also acts on a different receptor protein. Hence, CDA is related to stages after the establishment of the bacterium and to a PsrA independent regulatory pathway. Therefore, its main function will be to disintegrate the biofilm and activate systemic dispersal mechanisms (toward other organs of the host) such as swarming, but also to inactivate the T3SS (**Figure 4B**) that requires a high energy cost for its formation.

We can conclude that our data supports growing evidence that *in vitro* studies may be inadequately simulating *in vivo* conditions (Díaz-Pascual et al., 2017). Although SAFA C12-18 exhibited anti-virulence properties *in vitro*, their role *in vivo* is that of environmental signals for the activation of *P. aeruginosa* virulence factors during specific growth phases. Therefore, we suggest that lauric and myristic acids in the microenvironment function as signal molecules for the global regulation of virulence genes in the stationary phase through an interaction with PsrA.

## Data Availability Statement

The raw data supporting the conclusions of this article will be made available by the authors, without undue reservation.

## Ethics Statement

The animal study was reviewed and approved by Hospital Infantil de México-Federico Gómez (HIM2018-002).

## Author Contributions

MJ-R, IC-J, RG-C, and BG-P conceived and designed the experiments. MJ-R, HC-L, and MD-G carried out the experiments. MJ-R, IC-J, RG-C, and BG-P analyzed the data. MJ-R and IC-J wrote the main manuscript. BG-P, MM-V, JR-C, and RS-H critically reviewed the manuscript. All authors contributed to the article and approved the submitted version.

## Funding

This work was supported by grant from Scientific Development Projects for Solving National Problems/CONACyT Mexico no. 2015-01-402. MMJ-R thanks CONACYT for her master scholarship (907614 ). HC-L thanks CONACYT for her doctoral scholarship (449277). IC-J was supported by Cátedras- CONACyT program. RG-C is funded by CONACYT grant CB-2017-2018 number A1-S-8530 and BG-P is funded by CONACYT grant CB-284081.

## Conflict of Interest

The authors declare that the research was conducted in the absence of any commercial or financial relationships that could be construed as a potential conflict of interest.
