# Supplementary material for: Tetradecanoic Acids With Anti-Virulence Properties Increase the Pathogenicity of Pseudomonas aeruginosa in a Murine Cutaneous Infection Model
Source: Front Cell Infect Microbiol. 2021 Jan 27;10:597517. doi: 10.3389/fcimb.2020.597517 (PMC7876447; doi:10.3389/fcimb.2020.597517)
Supplement: Supplementary file 1 [file DataSheet_1.docx]

Supplementary Material

## Supplementary Tables

# Supplementary Table 1. Strains of *P. aeruginosa* used in this study

| Strains | Features | Reference |
| --- | --- | --- |
| PA14 WT | Reference strain isolated from a burn patient. | Liberati, Urbach & Miyata, *et al*., 2006. |
| Δ*lasR*/Δ*rhlR* | PA14 mutant strain with Las and Rhl system disrupted. | Dekimpe & Dèziel, 2009. |
| Δ*pscC* | PA14 mutant strain with *pscC* gene of T3SS disrupted. | Liberati, Urbach & Miyata, *et al*., 2006. |

(Liberati et al., 2006; Dekimpe and Déziel, 2009)

# Supplementary Table 2. Influence of lauric and myristic acids on the pathogenicity of mutant strains

| Treatments | Abscess area  (mm^2^_,_ mean + S.E.) | Necrotic area  (mm^2^_,_ mean + S.E.) | Inoculation area  (*log* CFU/g) | Liver  (*log* CFU/g) | Survival (%) |
| --- | --- | --- | --- | --- | --- |
| PA14 WT | 22.4 + 22.4 | 8.3 + 6.9 | 4.5 + 2.4 | 0.8 + 0.8 | 50 |
| *ΔlasR/ΔrhlR* | 9.6 + 9.6 | 0.3 + 0.3 | 0 + 0* | 0 + 0 | 100 |
| *ΔpscC* | 5.9 + 5.9* | 0 + 0* | 0 + 0* | 0.7 + 0.7 | 100 |
| Lauric acid (200 μM) | | | | | |
| *ΔlasR/ΔrhlR* | 1.4 + 1.4* | 0.8 + 0.8 | 1.1 + 1.1 | 0 + 0 | 100 |
| *ΔpscC* | 0 + 0* | 0 + 0* | 0 + 0* | 0 + 0 | 100 |
| Myristic acid (200 μM) | | | | | |
| *ΔlasR/ΔrhlR* | 0 + 0* | 1.7 + 0.8 | 3.3 + 1.3 | 0.6 + 0.6 | 83.3 |
| *ΔpscC* | 0 + 0* | 0 + 0* | 0 + 0* | 0 + 0 | 100 |

The abscess area was determined at 24 h and the necrotic area at 48 h. Injured tissues and liver were harvested 96 h after infection, homogenized, and the number of *log* CFU/g of tissue was calculated. The experiments were carried out with groups of five and six mice in a single trial. Significant difference from the wild type (**α* < 0.05), Mann-Whitney test. Δ*lasR*/Δ*rhlR* = QS mutant. Δ*pscC* = SST3 mutant. DMSO was also added to the untreated controls in an amount equivalent (final concentration ≤ 2.5%) to that of the treatment groups.

## Supplementary Figures


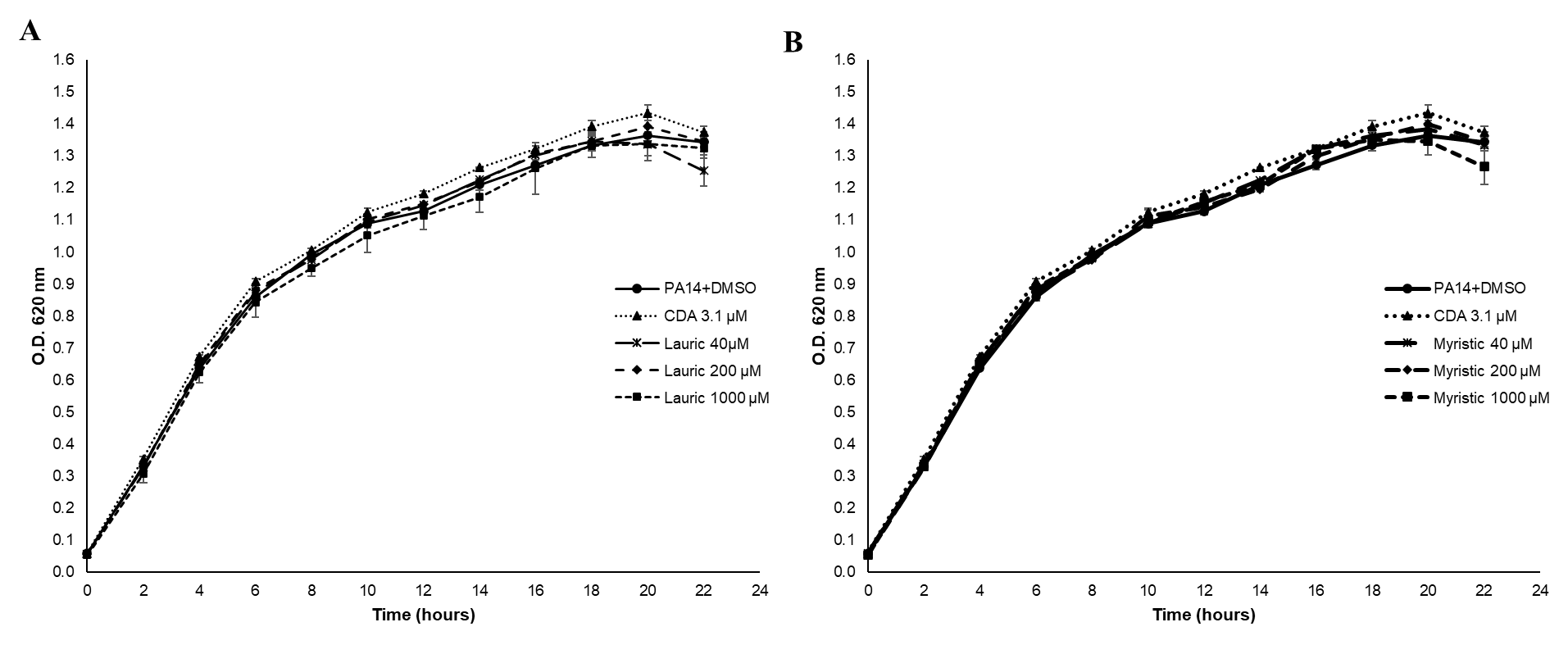
**Supplemental Figure 1.** Effect of FA on the growth of PA14. A) lauric acid and B) myristic acid. CDA, *cis*-2-decenoic acid.

**
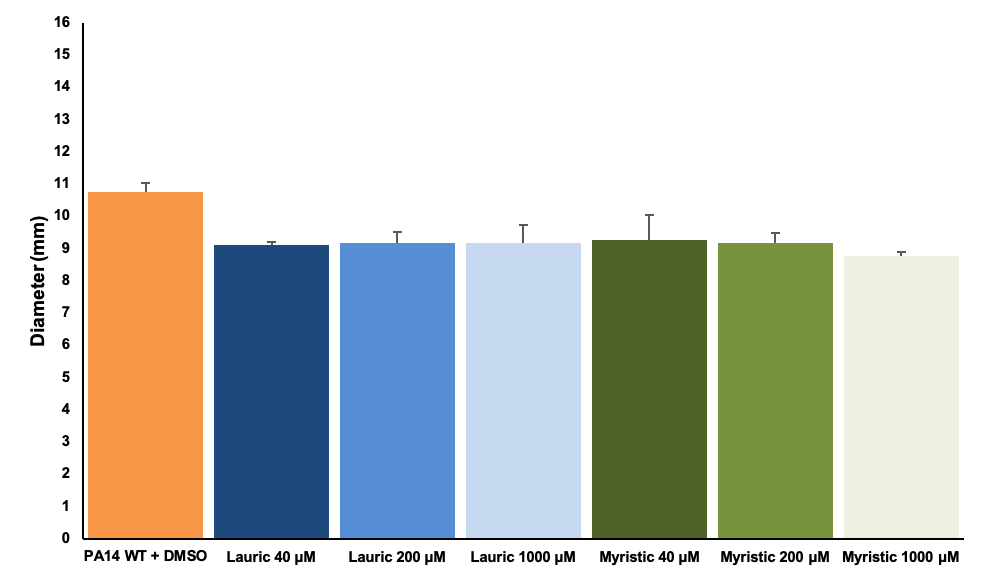
**

**Supplemental Figure 2.** Effect of SAFA on hemolytic activity from *P. aeruginosa.* The results shown are the means of three repetitions and the standard error of the mean. Significant difference from the wild type (**α* < 0.05), Student´s t-test.


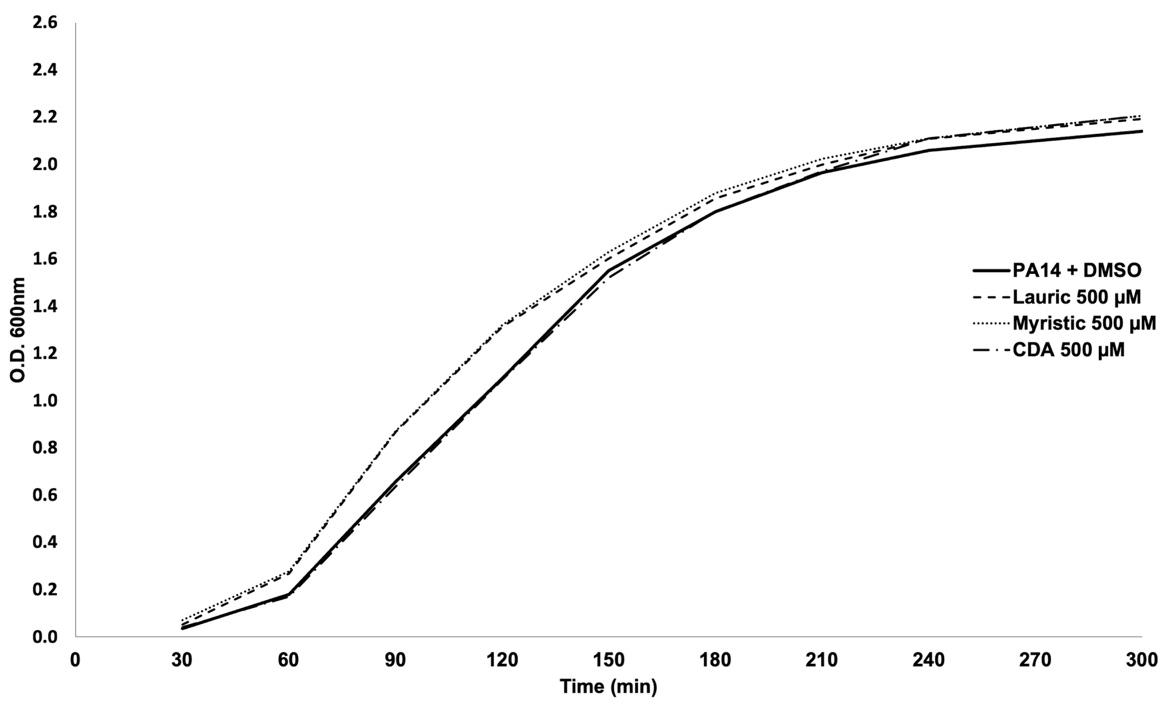


**Supplemental Figure 3.** Effect of fatty acids and *cis*-2-decenoic acid (CDA) on the growth of *P. aeruginosa* in the secretion assays of the ExoU protein.


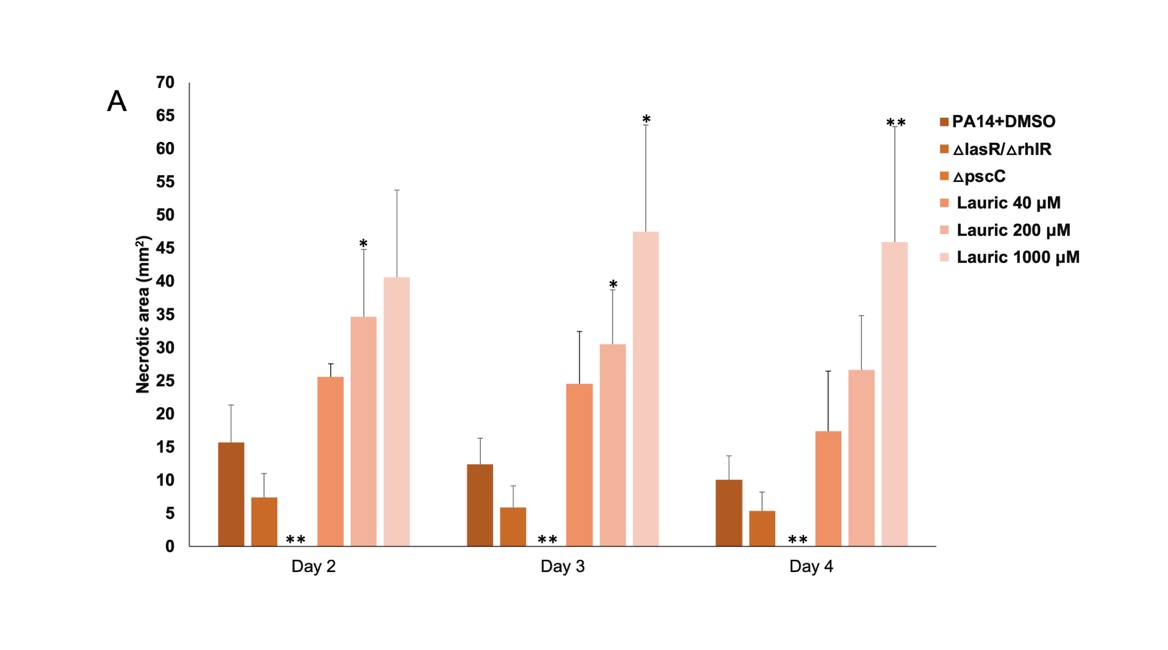

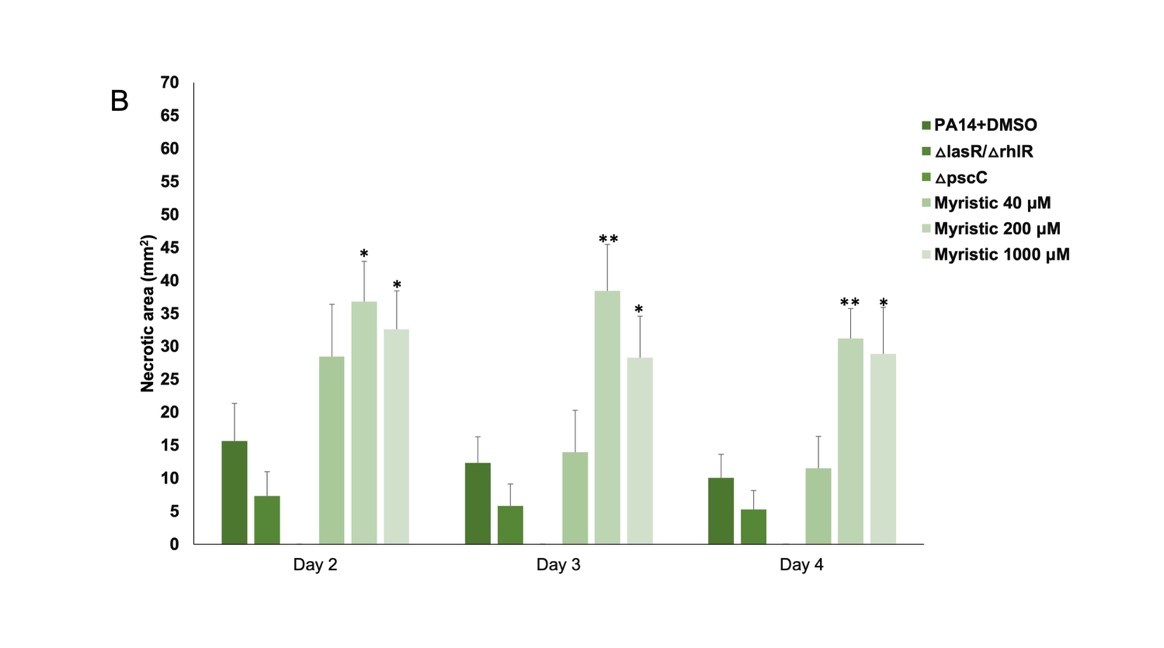

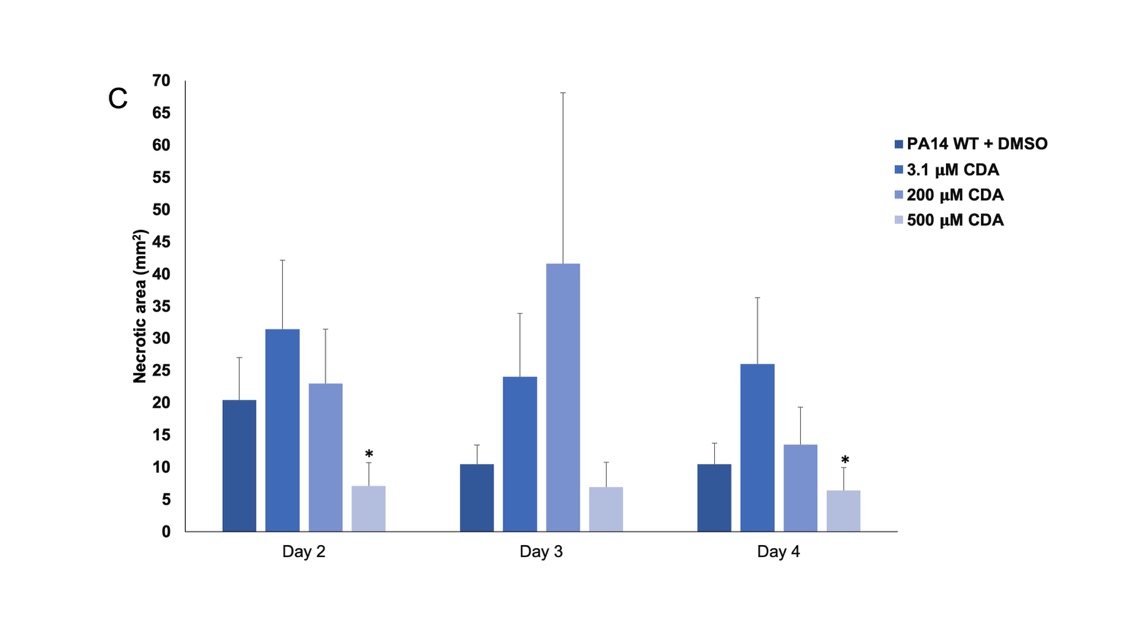


**Supplemental Figure 4.** Formation of necrotic area in animals by the co-administration of *P. aeruginosa* PA14 with fatty acids and *cis*-2-decenoic acid. **A**) lauric acid, **B**) myristic acid and **C**) CDA. Significant differences from the wild type (**a* < 0.05, ***a* < 0.01; Mann-Whitney test). Δ*lasR*/Δ*rhlR*, QS mutant. Δ*pscC*, T3SS mutant were found. DMSO was also added to the untreated controls in an amount equivalent (final concentration ≤ 2.5%) to that of the treatment groups.

**
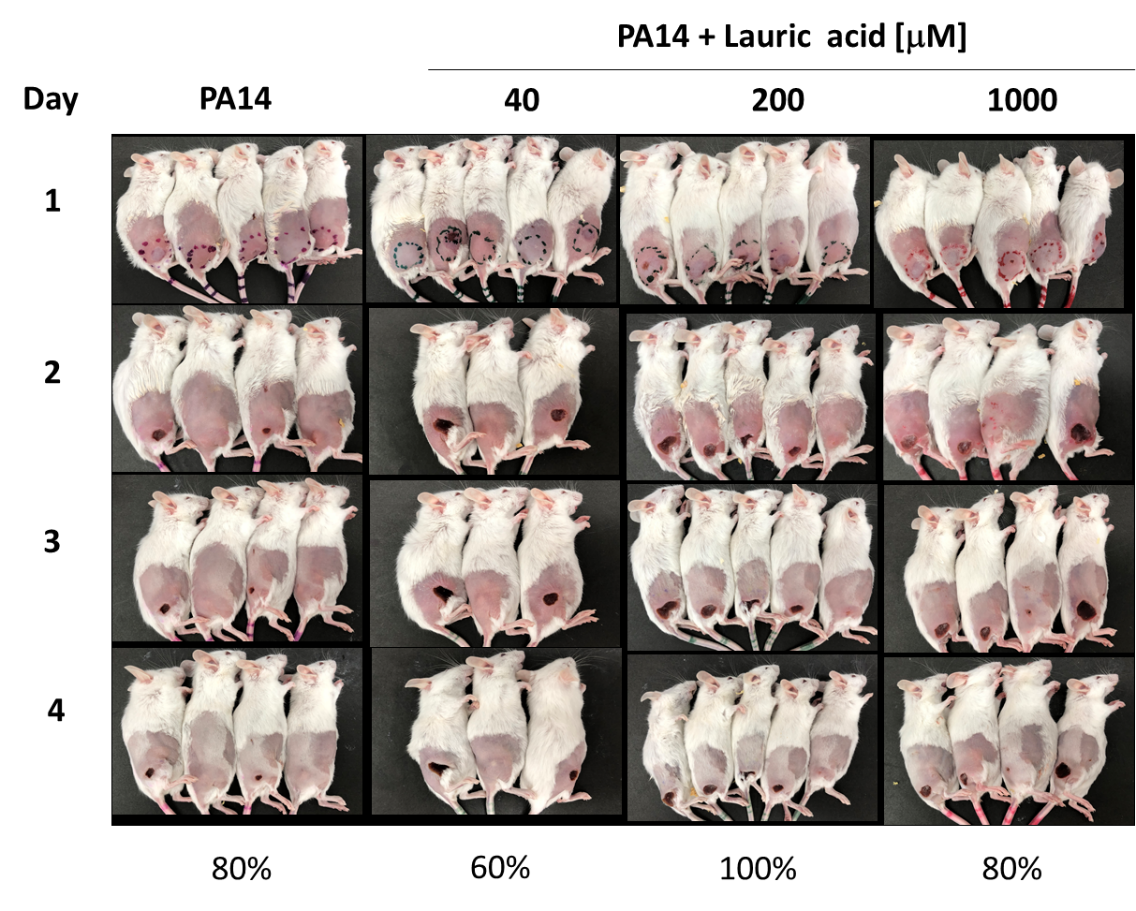
**

**Supplemental Figure 5.** Experiment 1: Inflammation and necrosis in mice inoculated with *P. aeruginosa* PA14 + lauric acid. At the bottom of the image the survival percentages for the fourth day are shown. (n = 5). PA14 is a control without fatty acid to which DMSO was added in an equivalent quantity (final concentration ≤ 2.5%) to that of the groups inoculated with lauric acid, which was previously dissolved in this organic solvent.


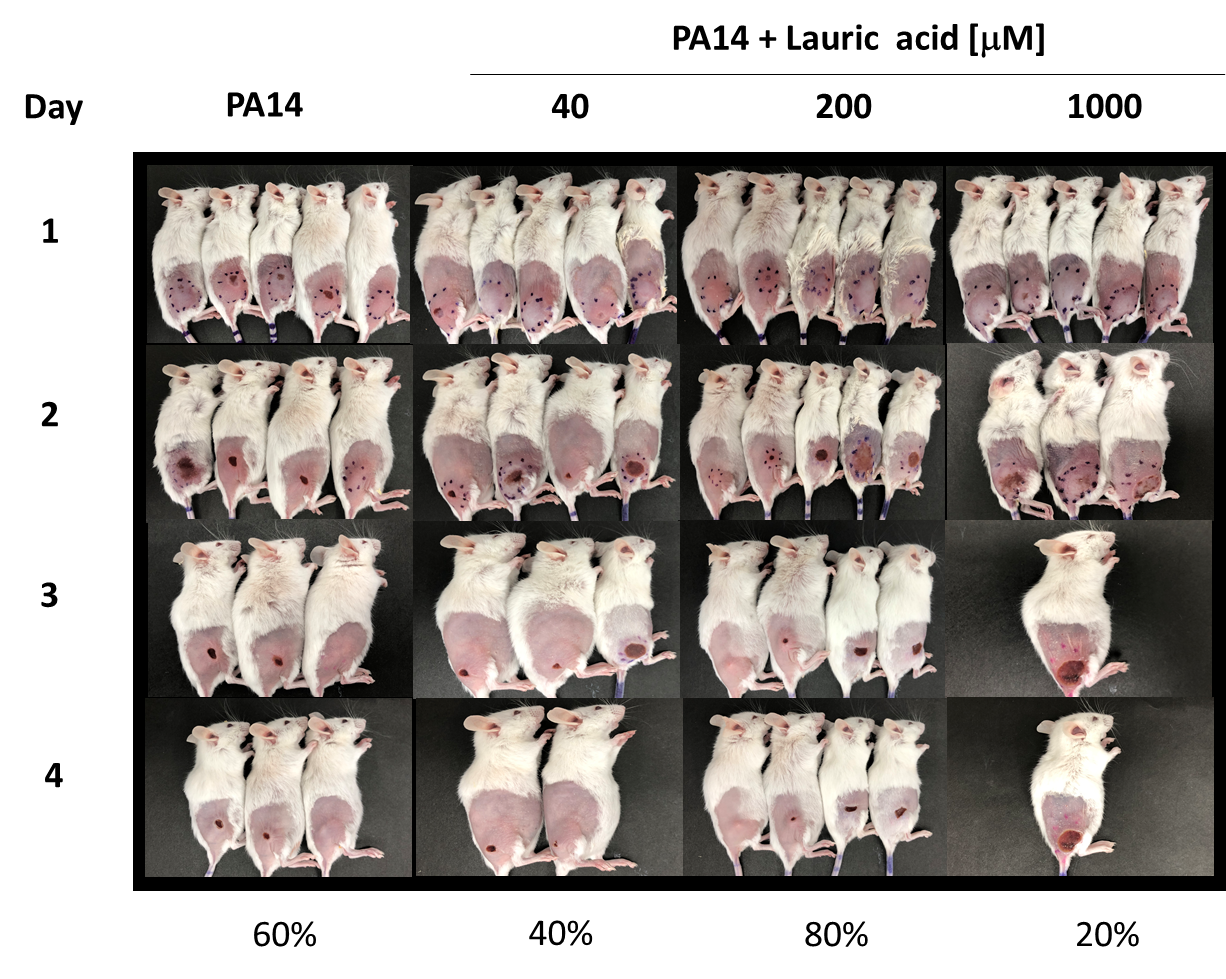


**Supplemental Figure 6.** Experiment 2: Inflammation and necrosis in mice inoculated with *P. aeruginosa* PA14 + lauric acid. At the bottom of the image the survival percentages for the fourth day are shown. (n = 5). PA14 is a control without fatty acid to which DMSO was added in an equivalent quantity (final concentration ≤ 2.5%) to that of the groups inoculated with lauric acid, which was previously dissolved in this organic solvent.


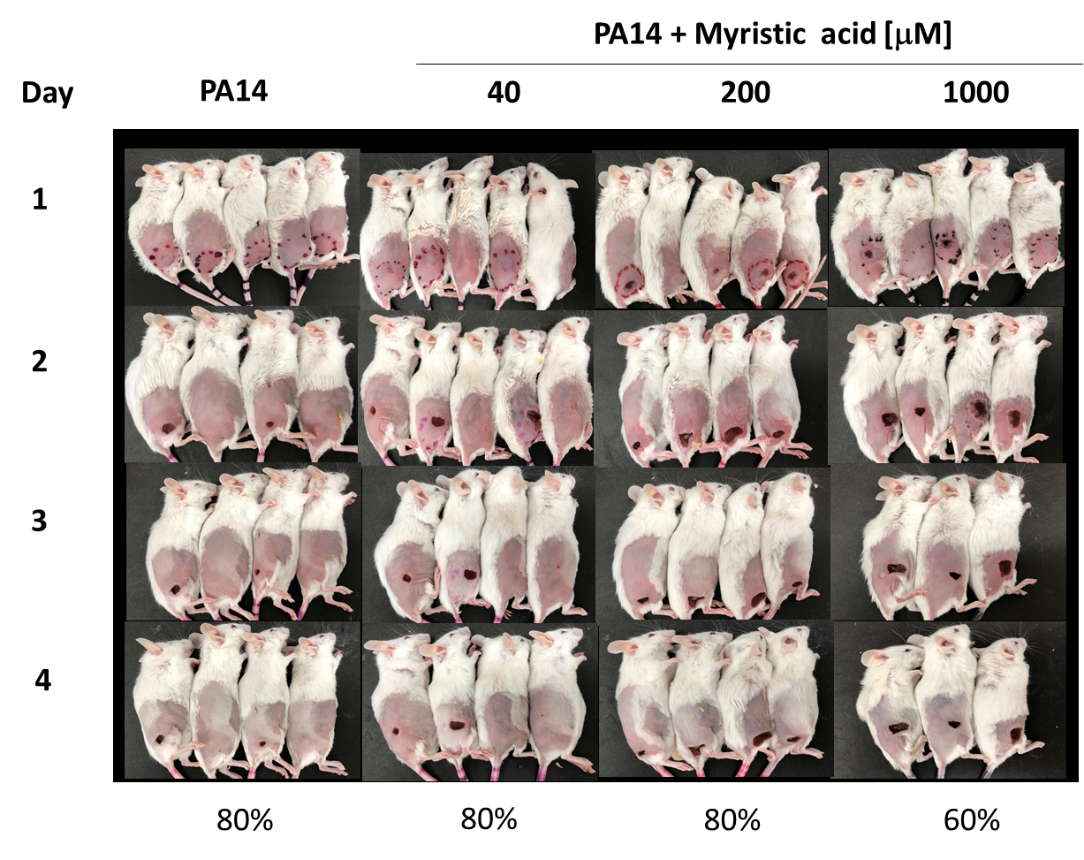


**Supplemental Figure 7.** Experiment 1: Inflammation and necrosis in mice inoculated with *P. aeruginosa* PA14 + myristic acid. At the bottom of the image the survival percentages for the fourth day are shown. (n = 5). PA14 is a control without fatty acid to which DMSO was added in an equivalent quantity (final concentration ≤ 2.5%) to that of the groups inoculated with myristic acid, which was previously dissolved in this organic solvent.


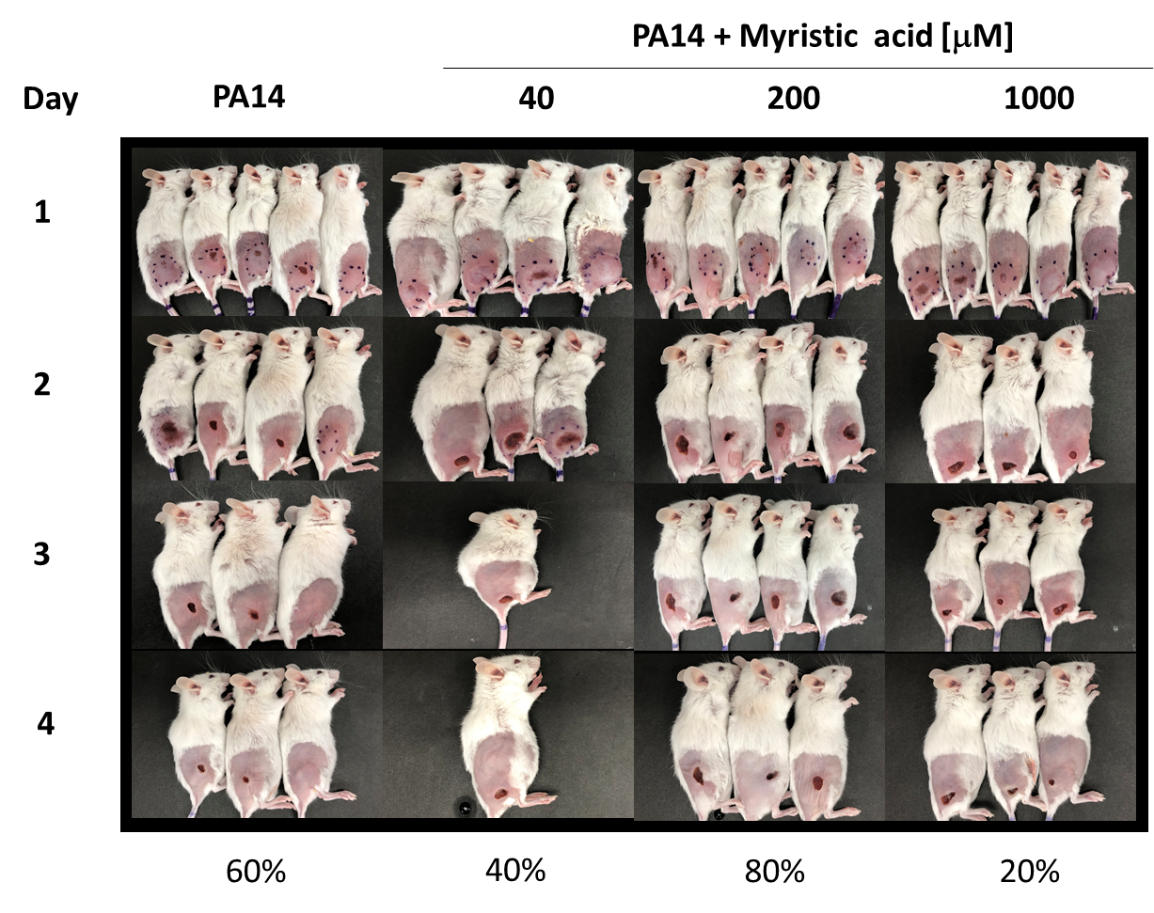


**Supplemental Figure 8.** Experiment 2: Inflammation and necrosis in mice inoculated with *P. aeruginosa* PA14 + myristic acid. At the bottom of the image the survival percentages for the fourth day are shown. (n = 5). PA14 is a control without fatty acid to which DMSO was added in an equivalent quantity (final concentration ≤ 2.5%) to that of the groups inoculated with myristic acid, which was previously dissolved in this organic solvent.


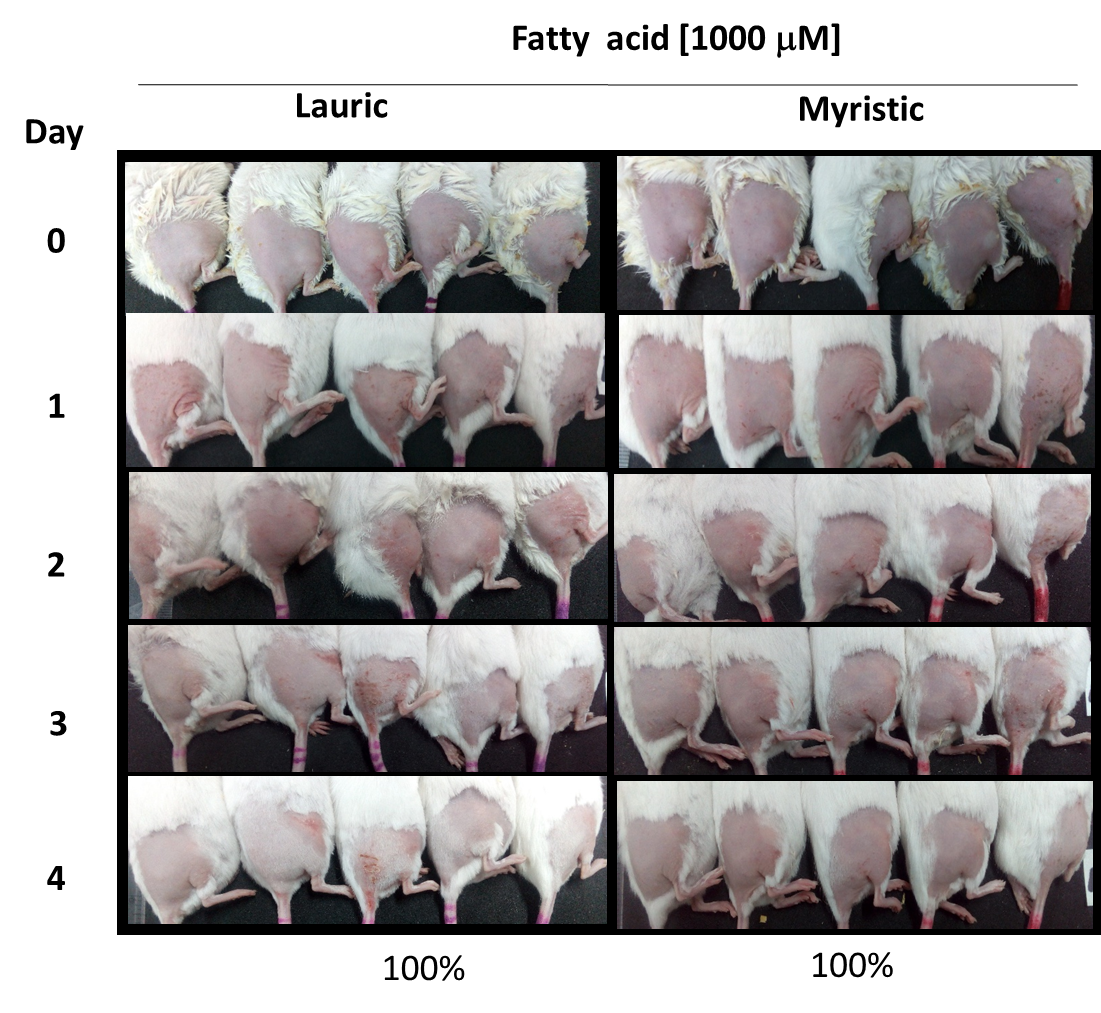


**Supplemental Figure 9.** Effect of lauric and myristic fatty acids at 1000 μM without bacteria. No inflammation or formation of necrotic areas was observed during the four days of experimentation. (n = 5).


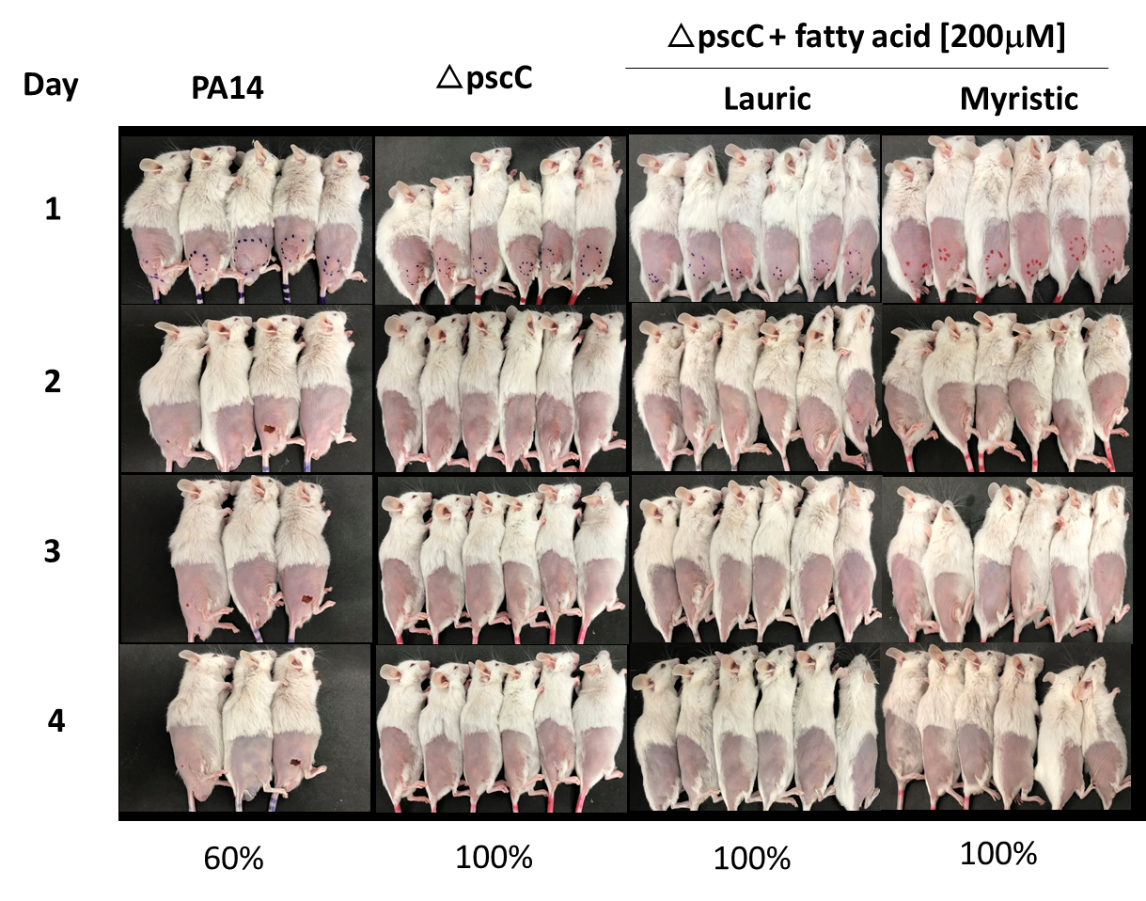


**Supplemental Figure 10.** Inflammation and necrosis in mice inoculated with the mutant strain ΔpscC + fatty acids. At the bottom of the image the survival percentages for the fourth day are shown. (n = 5 and 6). PA14 and ΔpscC are controls without fatty acid to which DMSO was added in an equivalent amount (final concentration ≤ 2.5%) to that of the groups inoculated with lauric and myristic acid.


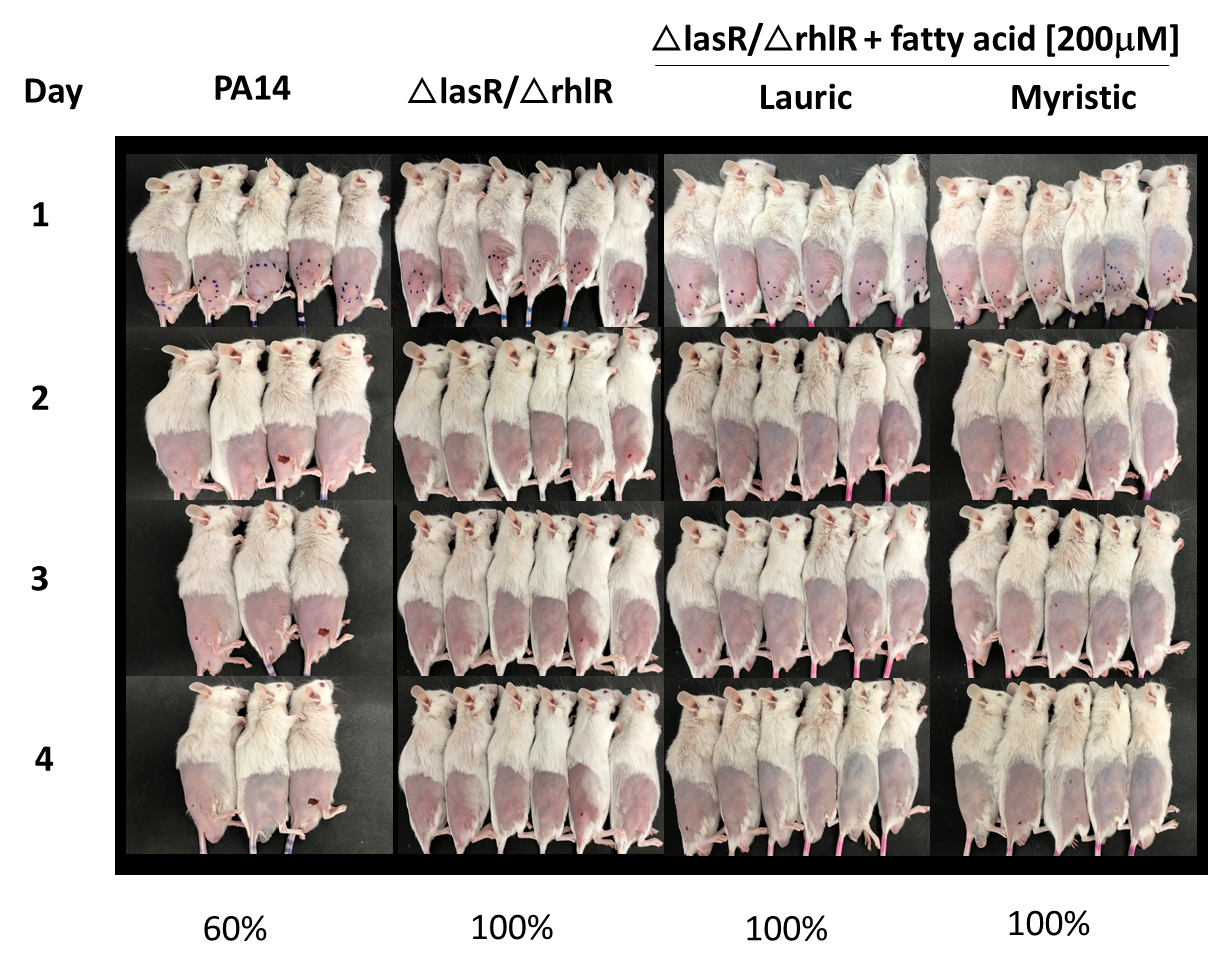


**Supplemental Figure 11.** Inflammation and necrosis in mice inoculated with the mutant strain Δ*lasR*/Δ*rhlR* + fatty acids. At the bottom of the image the survival percentages for the fourth day are shown. (n = 5 and 6). PA14 and Δ*lasR*/Δ*rhlR* are controls without fatty acid to which DMSO was added in an equivalent amount (final concentration ≤ 2.5%) to that of the groups inoculated with lauric and myristic acid.


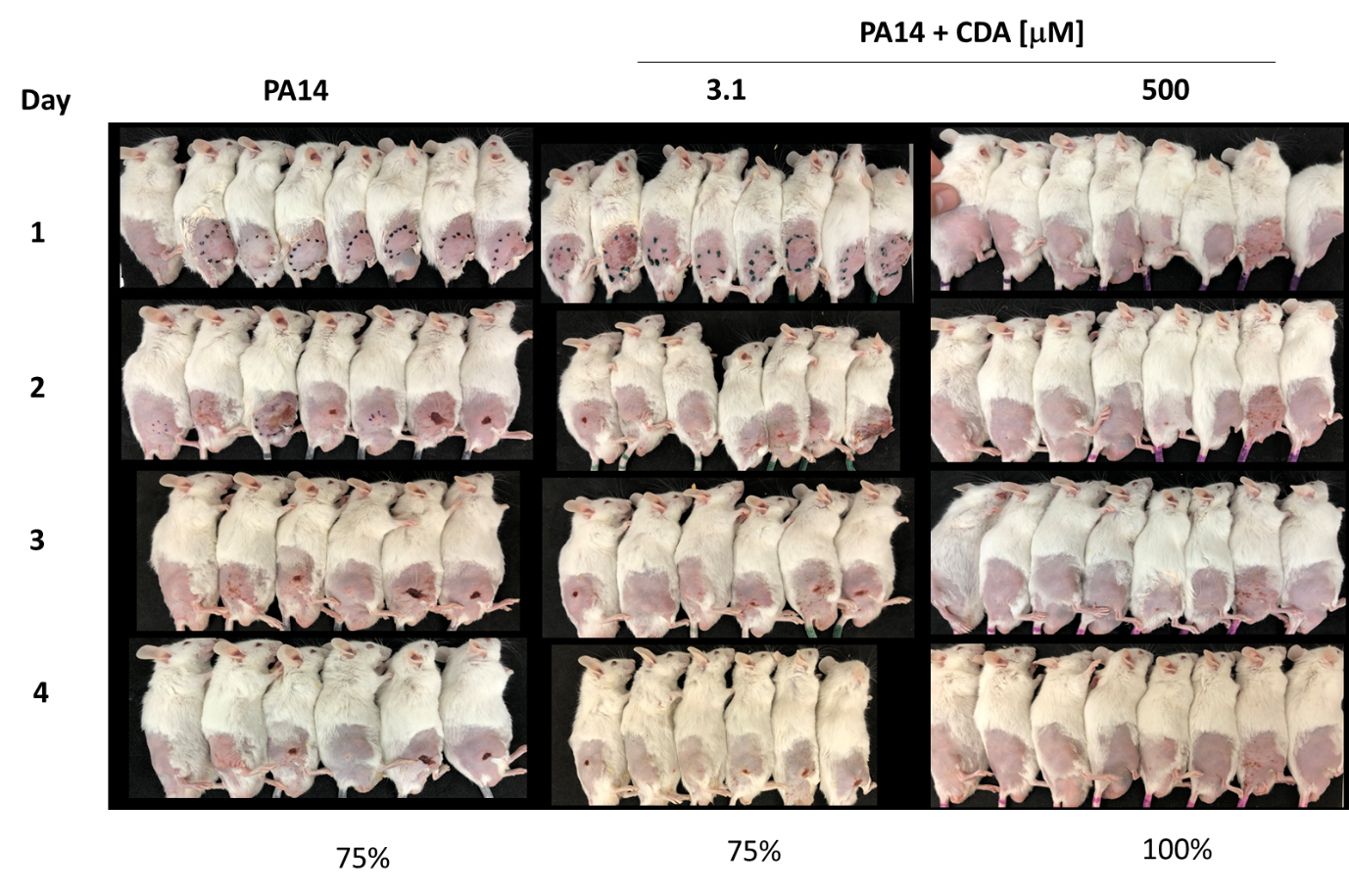


**Supplemental Figure 12.** Representative experiment: inflammation and necrosis in mice inoculated with PA14 + CDA. At the bottom of the image the survival percentages for the fourth day are shown. (n = 8). PA14 is a control without fatty acid to which DMSO was added in an equivalent quantity (final concentration ≤ 2.5%) to that of the groups inoculated with CDA, which was previously dissolved in this organic solvent.
